# Connubimur

**Published:** 1867-02

**Authors:** T. O. Edwards

**Affiliations:** Lancaster, Ohio


"*Connubimur.*"—By T. O. Edwards, M.D., of Lancaster, Ohio.

Allow me a small space to endeavor to settle the great controversy now existing in relation to the true meaning of the words contagion and infection. Some regard them as synonymous, others as signifying things of very different meaning. "Who shall decide when doctors disagree? I cannot suppress the belief that these two words did originally denote ideas very unlike each other, and that at this time they ought not to be confounded.

There is scarcely an instance of two words in the English or Latin, or any other language possessing the same critical meaning. Though in common speech they may be employed as convertible terms; yet they are always found, on nice examination, to have a plain and sensible difference. The books of rhetoric and belles letters inform us wherein "pride" differs from "vanity;" how "fatigue" is distinguished from "uneasiness," and by what means "delight" varies from "pleasure," with hundred other examples, these, though in common acceptation reputed to be synonymous words, are in reality very far from each other in true significance.

What happens in the language of common life occurs also in the dialect of medicine; words reputed by many to be quite alike, are known by the correct and learned to intend things quite remote in their meaning. Thus "lues," "pestis," "contagium," and "infectio" have been used by many as words of signification so nearly alike, that in glossaries and lexicons they have been familiarly employed one for the other. This is a mistake. Lues seems to be derived from "*luo*" "*to pay the cost,*" "*make atonement,*" *or* "*suffer punishment for a crime or fault.*" Hence *luere pœnas* signifies to suffer the penalty for an omission or breach of duty. And for the same reason "lues" is employed to mean any distemper brought on through or by a violation of moral duty; particularly as it applies to the disease consequent upon scortatory love; which has been termed emphatically "lues veneria"—the malady incident to prostituted embraces. Such is the literal and original meaning of the word; but like other words it acquired afterwards a greater latitude of signification. Thus Claudean, the poet, writes: "Hinc hominem, pecudumque lues, hinc pestifer aer," alluding to the sufferings of men and cattle; and Virgil goes a step further and extends the idea to trees and corn; "Arboribusque satisque lues, et lethifer annus."

Whether pestis is derived from perco, to perish or to die; or comes from the Greek word "paschat," imputing to spoil or pillage, seems not now necessary to be disputed. It is sufficient for the present purpose to show that whether the former or the latter etymology be adopted "pestes" means something ruinous and destructive, generally applying to such calamities that cannot be prevented by human foresight. Pestilence is therefore coupled by Virgil with the anger of the Gods.

"Pestis et ira decorum stygiis sese extulit undis."

And there is classical authority for the following:

"Me cruciat a sævo pestis violenta veneno."

Showing that the word originally signified almost any unavoidable distress; and, in a limited sense, applied particularly to disastrous sickness and endemic distemper.

No person doubts that *contages*, *contagium*, and *contagion* are fair derivations from *contingo*, to touch or be in contact—their primative sense doubtless was, "diseases communicable by approximation of skin to skin." Hence these expressions apply peculiarly to *gregarious* animals, as sheep, cattle, and the human species. Creatures of these kind huddling and mingling together, associating while they feed and when they lie down to rest, are remarkably prone to catch diseases by *contact*. In this strain, Melibœus assures Tityrus (Virgil, Ecl. i. v. 51) that the noxious contagion of his neighbor's flock shall do no injury to his:—

"Nec mala vicini pecoris *contagio* laedent."

The forms of expression, when either of these words is used, are adapted to give an idea of something creeping, or passing off from one person to another, thus:—

"Dira per in incantum *serpunt* contagia vulgus;"

And again:—

"Dira per omnes

Manebant populus fædi contagio morbi."

And also, "*Invadunt totum contagia morbida regnum.*" From these authorities, it appears plain that the popular and obvious meaning of contagion could scarcely be misunderstood, since it respected merely that class of disorders among men and brutes which were imparted from one to another by contact, as they fed, slept, played, and associated together.

Contagion, therefore, is that peculiar, morbid poison which is prepared in the bodies of living animals, especially of those which flock and huddle together in crowds, and is wiped off, or is communicated by touch from those contaminated to those too nearly approaching them. Such was the common sense of mankind, while it remained unwarped by prejudice and forced meanings. It is a pity that such an unlettered though common sense was ever departed from.

Infection was certainly derived from "*inficio.*" This word was of very various and questionable meaning among Romans; being composed of *in*, negative, and *facio*, to do, signifying to undo; or, rather, to violate, to corrupt, to taint, or to tincture anything. To be a little more particular, the verb *inficio* expressed that property of an agent, by which it imbued the substance on which it acted, manifestly and glaringly changed its qualities, and altered it materially. The substance or thing so altered, was said to be infected. One of the most frequent and distinguishable cases of infection was the change white cloth underwent in dyeing. The coloring material, or the dye-stuff, was the agent, and the changing or vitiating the white color by its tinging property, was said to infect or stain the fabric; its whiteness was alleged to be overcast, polluted, spoiled, or undone. So when the clear and fine atmosphere was vitiated by mixtures of noxious vapors and poisonous gases, extricated from corrupting bodies, it was declared to be infected. The pure air, like the white cloth, had acquired a foreign tincture; and this effect, wrought upon the respirable and healthy atmosphere by the adventitious material with which it was charged, was, in a figurative sense, denominated *infection*. The air so vitiated or corrupted, so different in its constitution and so altered, had become infected or *infectus*, that is, undone, or spoiled for the purpose, of its primitive or ordinary purpose. Air thus vitiated, was infected air. In like manner, a healthy animal might become infected by coming into an atmosphere of such a tincture, or so corrupted. An external agent of this kind could infect or undo the healthy frame. When this act of undoing, or, in other words, of unfitting it for the performance of its accustomed and useful functions was accomplished, the constitution was affirmed to be infected. As was observed in a preceding paragraph, infection was, in one of its senses, but another term for dyeing or imparting colors. In many forms of distempers exerted by pestilential air, there was observed, in addition to the other symptoms of debility, etc., a remarkable change of complexion. In many cases, the patients looked almost as if they had been dyed, or colored by some tinging material. This confirmed the notion, that infection having penetrated the body and wrought a change, as evident to the eyes of others as uncomfortable to the individual afflicted. By this process of reasoning, it seems to have been accepted and understood that a white garment put into dye-stuff, pure air exposed to septic exhalations, and a healthy animal acted upon by a pestilential atmosphere, were all examples of "infection."

The term was applied to another case. When anything noxious was added to wholesome drink, it was declared to be infected, that is, to be undone or spoiled for the natural and intended purpose of quenching thirst. Hence the verse can be interpreted, "Pocula si quando sævæ *infecere* novercæ." These are popular not scientific distinctions. There are but two operations in nature for preparing and engendering noxious fluids. The former is accomplished by the instrumentality of the living vascular action of animals and, it may be, of vegetables; the latter, the result of putrefaction, and, consequently, out of the power of life. To signify these two grand natural processes, there ought to be two suitable and technical terms. Contagion and infection, when properly understood, denote them, but, as expressed in common medical language, they are too frequently regarded as synonymous. *That vitiated product of living vascular action* which can excite in a well person a disease like that by which itself was produced, and continue indefinitely to do so after being transferred from one body to another, will be and is properly denominated contagion; and lues, vaccinia, measles, and small-pox are examples of it. On the other hand, that *venomous offspring of putrefaction* going on in some of the kinds of organic matter *after death*, a *separation from the living frame*, which disorders the healthy functions without being specifically communicable, and without the power of communicating itself, will be called *infection;* and typhus, dysentery, plague, and yellow fever will be given as instances. Having thus given early acceptation of these terms, I propose to continue the subject and apply them intelligibly to modern nomenclatures.

